# 4-(9-Anthryl)-2-methylbutyn-2-ol

**DOI:** 10.1107/S1600536808001542

**Published:** 2008-01-23

**Authors:** Irena Bylińska, Artur Sikorski, Wiesław Wiczk

**Affiliations:** a Faculty of Chemistry, University of Gdańsk, J. Sobieskiego 18, 80-952 Gdańsk, Poland

## Abstract

There are two mol­ecules in the asymmetric unit of the title compound, C_19_H_16_O. Neighbouring mol­ecules are linked through O—H⋯O hydrogen bonds into an *R*_4_^4^(8) ring motif. There are also C—H⋯π hydrogen and π–π inter­actions. The mol­ecules are either parallel to each other or are inclined at an angle of 12.5 (1)°.

## Related literature

For applications of this class of compounds, see: Bunz (2000 ▶); De Silva *et al.* (1999 ▶), Krasovitski & Bolotin (1988 ▶); O’Regan & Grätzel (1991 ▶); Schumm *et al.* (1994 ▶). For the use of ethynyl­anthracene derivatives in organic synthesis, see Wen *et al.* (2004 ▶); Xiao *et al.* (2007 ▶). For comparison bond dimensions of the anthracene skeleton, see: Cuffet *et al.* (2005 ▶); Elangovan *et al.* (2005 ▶). For the structure of 9,10-bis­(3-hydr­oxy-3-methyl-1-butyne)anthracene, see: Dang *et al.* (2002 ▶).
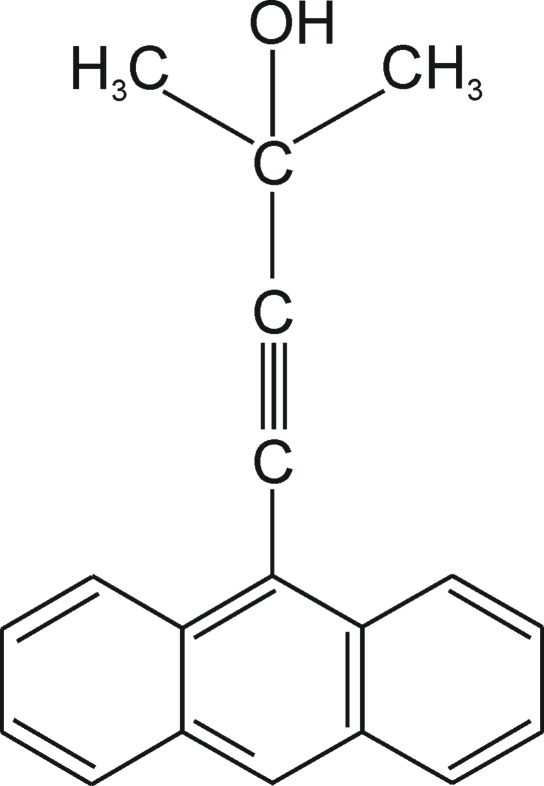


## Experimental

### 

#### Crystal data

C_19_H_16_O
*M* *_r_* = 260.32
Triclinic, 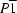

*a* = 9.995 (2) Å
*b* = 12.738 (3) Å
*c* = 12.905 (3) Å
α = 75.70 (3)°
β = 72.18 (3)°
γ = 68.84 (3)°
*V* = 1441.4 (7) Å^3^
*Z* = 4
Mo *K*α radiation
μ = 0.07 mm^−1^
*T* = 298 (2) K
0.60 × 0.20 × 0.10 mm

#### Data collection

Kuma KM-4 diffractometer
Absorption correction: none
5258 measured reflections
5011 independent reflections
2370 reflections with *I* > 2σ(*I*)
*R*_int_ = 0.019
3 standard reflections every 200 reflections intensity decay: 1.1%

#### Refinement

*R*[*F* ^2^ > 2σ(*F* ^2^)] = 0.051
*wR*(*F* ^2^) = 0.170
*S* = 0.98
5011 reflections
366 parameters
H-atom parameters constrained
Δρ_max_ = 0.25 e Å^−3^
Δρ_min_ = −0.34 e Å^−3^

### 

Data collection: *KM-4 Software* (Oxford Diffraction, 1995-2003 ▶); cell refinement: *KM-4 Software*; data reduction: *KM-4 Software*; program(s) used to solve structure: *SHELXS97* (Sheldrick, 2008 ▶); program(s) used to refine structure: *SHELXL97* (Sheldrick, 2008 ▶); molecular graphics: *ORTEPII* (Johnson, 1976 ▶); software used to prepare material for publication: *SHELXL97* and *PLATON* (Spek, 2003 ▶).

## Supplementary Material

Crystal structure: contains datablocks global, I. DOI: 10.1107/S1600536808001542/ng2418sup1.cif

Structure factors: contains datablocks I. DOI: 10.1107/S1600536808001542/ng2418Isup2.hkl

Additional supplementary materials:  crystallographic information; 3D view; checkCIF report

## Figures and Tables

**Table 1:** Hydrogen-bond geometry (Å, °)

*D*—H⋯*A*	*D*—H	H⋯*A*	*D*⋯*A*	*D*—H⋯*A*
O18—H18⋯O38^i^	0.82	2.01	2.726 (3)	145
O38—H38⋯O18^ii^	0.82	2.06	2.766 (3)	145

Symmetry codes: (i) 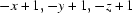; (ii) 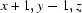.

**Table 2:** C—H⋯π interactions (Å,°) *Cg*1 is the centroid of the C5/C10–C14 ring and *Cg*2 is the centroid of the C1–C4/C12/C11 ring.

*X*	H	*J*	H⋯*J*	*X*⋯*J*	*X*—*I*⋯*J*
C19	H19*C*	*Cg*1^iii^	2.87	3.810 (4)	167
C20	H20*B*	*Cg*2^iii^	2.76	3.703 (4)	168
C24	H24	*Cg*2^iv^	2.64	3.472 (3)	149
C25	H25	*Cg*1^iv^	2.92	3.794 (3)	157

Symmetry codes: (iii) 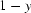, 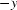, 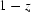; (iv) 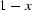, 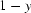, 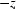.

**Table 3:** π–π interactions (Å,°) *Cg*3 is the centroid of the C25/30–C34 ring and *Cg*4 is the centroid of the C26-C29/C33/C34 ring. The dihedral angle is that between the planes of the rings *CgI* and *CgJ*. The interplanar distance is the perpendicular distance of *CgI* from ring *J*. The offset is the perpendicular distance of ring *I* from ring *J*.

*CgI*	*CgJ*	*Cg*⋯*Cg*	Dihedral angle	Interplanar distance	Offset
3	4^v^	3.794 (2)	1.2	3.370 (2)	1.336 (2)
4	3^v^	3.794 (2)	1.2	3.404 (2)	1.464 (2)

Symmetry code: (v) 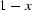, 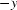, 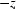.

**Fig. 1.**
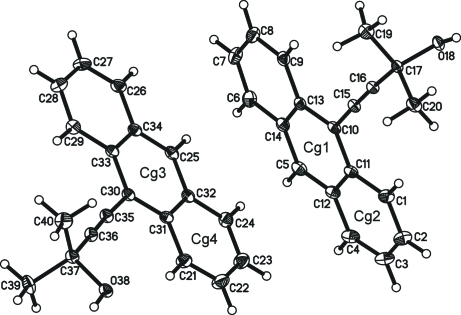
The molecular structure of the title compound showing the atom-labelling scheme. Displacement ellipsoids are drawn at the 25% probability level and H atoms are shown as small spheres of arbitrary radii. Cg1, Cg2, Cg3 and Cg4 denote the ring centroids.

**Fig. 2.**
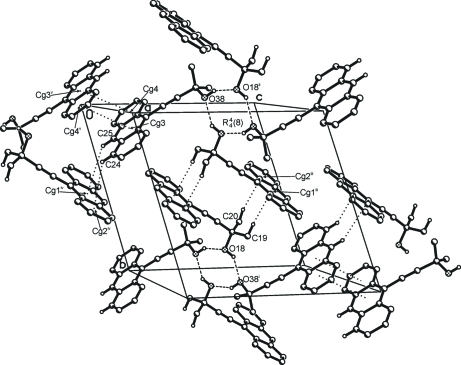
The arrangement of the ions in the unit cell, viewed along the c axis, showing R44(8) ring motifs. The O—H···O interactions are represented by dashed lines, and C—H···π and π-π interactions by dotted lines. H atoms not involved in interactions have been ommited. [Symmetry codes: (i) 1 - x, 1 - y, 1 - z; (ii) 1 + x, -1 + y, z; (iii) 1 - x, -y, 1 - z; (iv) 1 - x, -y, -z; (v) 1 - x, -y, -z.]

## supplementary crystallographic information

### Comment

Recently there is a need for molecules containing triple bond because of their electroconductive, magnetic and nonlinear optical properties. This class of the compounds are used as molecular wires (Bunz, 2000), molecular scale logic gates (De Silva *et al.*, 1999), optical and microelectronic devices (Schumm *et al.*, 1994), sensors (Krasovitski & Bolotin, 1988) and molecular photovoltaic cells (O'Regan & Grätzel, 1991)). Ethynylanthracene derivatives are substrates in many synthesis (Wen *et al.*, 2004; Xiao *et al.*, 2007). 2-Methyl-3-butyn-2-ol is an alternative protecting group for (trimethylsilyl)acetylene, more useful in reaction carried at higher temperature (because of its higher boiling point) as well as giving acetylene derivatives containing various substituents in homo- and heterocoupling. Because of that we use this protecting group to synthesize acetylene derivatives to study influence of the aromatic subtituent size on the photophysical properties of the compounds in search for organic material with extended π system, characterized by high fluorescence quantum yield. As an intermediate in the synthesis of anthracene derivative, 9-(3-hydroxy-3-methyl-1-butyne)anthracene was isolated and its crystal structure was determined.

Parameters characterizing the geometry of the anthracene skeleton are typical of anthracene-based derivatives (Cuffet *et al.*, 2005; Elangovan *et al.*, 2005).

In the crystal, the asymmetric unit consists of two molecules of the title compound (Fig. 1) which crystallizing in the triclinic crystal system, in P -1 space group, as well as 9,10-bis(3-hydroxy-3-methyl-1-butyne)anthracene (Dang *et al.*, 2002).

In the crystal structure, neighbouring molecules are linked through O—H···O hydrogen bond forming *R*_4_^4^(8) ring motif (Table 1 and Fig. 2). Molecules which forming this motif are linked by C—H···π hydrogen bonds (Table 2 and Fig. 2) or π-π interactions (Table 3 and Fig. 2). In the packing, the anthracene moieties are either parallel or inclined at an angle of 12.5 (1)°.

### Experimental

9-(3-hydroxy-3-methyl-1-butyne)anthracene has been synthesized by Sonogashira-Hagihara coupling from 9-bromoanthracene (10 mmol) and 2-methyl-3-butyn-2-ol (20 mmol) in DMF in the presence of Pd(PPh_3_)_4_ (0.0162 mmol) and Cu_2_I_2_ (0.13 mmol) as catalysts, triphenylphosphine (0.16 mmol), triethylamine (12 ml). The mixture was stirred at 333 K under argon atmosphere for 24 h. The reaction was monitored by TLC (petroleum ether-ethyl/acetate 10:1 *v*/*v*, *R*_f_=0.64; Merck Silica-gel plates (Kieselgel 60 F_254_)). When the reaction was completed the catalysts were filtered off, filtrate was poured into water and extracted with ethyl acetate. The brownish-red organic layer was dried over anhydrous MgSO_4_. The solvent was removed *in vacuo* giving a brownish-red oil. The crude was isolated by column chromatography on silica gel (Merck, Silica gel 60, 0.040–0.063 mm) using petroleum ether-ethyl/acetate (10:1 *v*/*v*) as an eluent and then crystallized from ethyl acetate to give yellow crystals (79% yield) [m.p. = 400–402 K].

### Refinement

All H atoms were positioned geometrically and refined using a riding model, with C—H distances of 0.93 Å and with *U*_iso_(H) = 1.2*U*_eq_(C) (C—H = 0.96 Å and *U*_iso_(H) = 1.5*U*_eq_(C) for the methyl group) and O—H distances of 0.82 Å and with *U*_iso_(H) = 1.5*U*_eq_(C).

### Crystal data

**Table:**

C_19_H_16_O	*Z* = 4
*M**_r_* = 260.32	*F*_000_ = 552
Triclinic, *P*1	*D*_x_ = 1.200 Mg m^−^^3^
Hall symbol: -P 1	Mo *K*α radiation λ = 0.71073 Å
*a* = 9.995 (2) Å	Cell parameters from 50 reflections
*b* = 12.738 (3) Å	θ = 2.2–25.0º
*c* = 12.905 (3) Å	µ = 0.07 mm^−^^1^
α = 75.70 (3)º	*T* = 298 (2) K
β = 72.18 (3)º	Block, white
γ = 68.84 (3)º	0.60 × 0.20 × 0.10 mm
*V* = 1441.4 (7) Å^3^	

### Data collection

**Table:**

Kuma KM4 diffractometer	*R*_int_ = 0.019
Radiation source: fine-focus sealed tube	θ_max_ = 25.0º
Monochromator: graphite	θ_min_ = 2.2º
*T* = 298(2) K	*h* = −11→11
θ/2θ scans	*k* = −14→14
Absorption correction: none	*l* = 0→15
5258 measured reflections	3 standard reflections
5011 independent reflections	every 200 reflections
2370 reflections with *I* > 2σ(*I*)	intensity decay: 1.1%

### Refinement

**Table:**

Refinement on *F*^2^	Hydrogen site location: inferred from neighbouring sites
Least-squares matrix: full	H-atom parameters constrained
*R*[*F*^2^ > 2σ(*F*^2^)] = 0.051	*w* = 1/[σ^2^(*F*_o_^2^) + (0.0961*P*)^2^ + 0.1391*P*] where *P* = (*F*_o_^2^ + 2*F*_c_^2^)/3
*wR*(*F*^2^) = 0.170	(Δ/σ)_max_ < 0.001
*S* = 0.98	Δρ_max_ = 0.25 e Å^−^^3^
5011 reflections	Δρ_min_ = −0.34 e Å^−^^3^
366 parameters	Extinction correction: SHELXL, Fc^*^=kFc[1+0.001xFc^2^λ^3^/sin(2θ)]^-1/4^
Primary atom site location: structure-invariant direct methods	Extinction coefficient: 0.036 (4)
Secondary atom site location: difference Fourier map	

### Special details

**Table:**

Experimental. no
Geometry. All e.s.d.'s (except the e.s.d. in the dihedral angle between two l.s. planes) are estimated using the full covariance matrix. The cell e.s.d.'s are taken into account individually in the estimation of e.s.d.'s in distances, angles and torsion angles; correlations between e.s.d.'s in cell parameters are only used when they are defined by crystal symmetry. An approximate (isotropic) treatment of cell e.s.d.'s is used for estimating e.s.d.'s involving l.s. planes.

### Fractional atomic coordinates and isotropic or equivalent isotropic displacement parameters (Å^2^)

**Table:**

	*x*	*y*	*z*	*U*_iso_*/*U*_eq_	
C1	0.6439 (3)	0.6352 (3)	0.2744 (3)	0.0703 (8)	
H1	0.6036	0.6730	0.3353	0.084*	
C2	0.7751 (4)	0.6414 (3)	0.2076 (3)	0.0873 (10)	
H2	0.8236	0.6839	0.2227	0.105*	
C3	0.8389 (4)	0.5848 (3)	0.1160 (3)	0.0878 (11)	
H3	0.9293	0.5897	0.0705	0.105*	
C4	0.7694 (3)	0.5232 (3)	0.0939 (3)	0.0767 (9)	
H4	0.8133	0.4850	0.0333	0.092*	
C5	0.5568 (3)	0.4561 (2)	0.1365 (2)	0.0635 (8)	
H5	0.6000	0.4182	0.0757	0.076*	
C6	0.3395 (4)	0.3939 (3)	0.1734 (3)	0.0737 (9)	
H6	0.3822	0.3552	0.1130	0.088*	
C7	0.2042 (4)	0.3938 (3)	0.2325 (3)	0.0816 (10)	
H7	0.1533	0.3565	0.2125	0.098*	
C8	0.1392 (4)	0.4496 (3)	0.3240 (3)	0.0763 (9)	
H8	0.0442	0.4507	0.3643	0.092*	
C9	0.2129 (3)	0.5021 (2)	0.3547 (2)	0.0626 (8)	
H9	0.1693	0.5364	0.4178	0.075*	
C10	0.4290 (3)	0.5670 (2)	0.3197 (2)	0.0519 (7)	
C11	0.5670 (3)	0.5725 (2)	0.2536 (2)	0.0549 (7)	
C12	0.6313 (3)	0.5154 (2)	0.1606 (2)	0.0571 (7)	
C13	0.3538 (3)	0.5062 (2)	0.2937 (2)	0.0497 (6)	
C14	0.4198 (3)	0.4511 (2)	0.1999 (2)	0.0565 (7)	
C15	0.3608 (3)	0.6290 (2)	0.4102 (2)	0.0528 (7)	
C16	0.3033 (3)	0.6851 (2)	0.4820 (2)	0.0508 (7)	
C17	0.2380 (3)	0.7569 (2)	0.5676 (2)	0.0473 (6)	
O18	0.1648 (2)	0.86882 (14)	0.52019 (15)	0.0609 (5)	
H18	0.1476	0.9139	0.5618	0.091*	
C19	0.1281 (3)	0.7122 (3)	0.6591 (2)	0.0733 (9)	
H19A	0.0470	0.7163	0.6316	0.110*	
H19B	0.0921	0.7572	0.7173	0.110*	
H19C	0.1748	0.6344	0.6869	0.110*	
C20	0.3575 (3)	0.7670 (3)	0.6081 (3)	0.0732 (9)	
H20A	0.4249	0.7962	0.5474	0.110*	
H20B	0.4098	0.6932	0.6413	0.110*	
H20C	0.3145	0.8178	0.6618	0.110*	
C21	0.8461 (3)	0.1228 (3)	0.0840 (3)	0.0745 (9)	
H21	0.8929	0.0796	0.1399	0.089*	
C22	0.9110 (4)	0.1911 (3)	0.0040 (3)	0.0928 (11)	
H22	1.0007	0.1960	0.0063	0.111*	
C23	0.8464 (4)	0.2547 (3)	−0.0822 (3)	0.0838 (10)	
H23	0.8933	0.3008	−0.1378	0.101*	
C24	0.7175 (4)	0.2495 (2)	−0.0849 (2)	0.0675 (8)	
H24	0.6750	0.2926	−0.1429	0.081*	
C25	0.5086 (3)	0.1752 (2)	−0.0037 (2)	0.0576 (7)	
H25	0.4649	0.2187	−0.0610	0.069*	
C26	0.2985 (3)	0.1032 (3)	0.0763 (3)	0.0709 (9)	
H26	0.2533	0.1480	0.0201	0.085*	
C27	0.2306 (4)	0.0359 (3)	0.1544 (3)	0.0865 (10)	
H27	0.1392	0.0347	0.1520	0.104*	
C28	0.2958 (4)	−0.0326 (3)	0.2399 (3)	0.0811 (10)	
H28	0.2477	−0.0790	0.2940	0.097*	
C29	0.4271 (3)	−0.0314 (3)	0.2436 (2)	0.0646 (8)	
H29	0.4701	−0.0785	0.3000	0.078*	
C30	0.6386 (3)	0.0447 (2)	0.1664 (2)	0.0496 (6)	
C31	0.7097 (3)	0.1152 (2)	0.0851 (2)	0.0527 (7)	
C32	0.6432 (3)	0.1807 (2)	−0.0028 (2)	0.0533 (7)	
C33	0.5030 (3)	0.0397 (2)	0.1639 (2)	0.0509 (7)	
C34	0.4362 (3)	0.1077 (2)	0.0770 (2)	0.0547 (7)	
C35	0.7051 (3)	−0.0211 (2)	0.2548 (2)	0.0573 (7)	
C36	0.7636 (3)	−0.0706 (2)	0.3274 (2)	0.0573 (7)	
C37	0.8328 (3)	−0.1305 (2)	0.4196 (2)	0.0559 (7)	
O38	0.9388 (2)	−0.07847 (15)	0.41664 (16)	0.0645 (6)	
H38	0.9901	−0.1179	0.4596	0.097*	
C39	0.9208 (4)	−0.2512 (2)	0.4034 (3)	0.0831 (11)	
H39A	0.9937	−0.2516	0.3347	0.125*	
H39B	0.8560	−0.2911	0.4026	0.125*	
H39C	0.9690	−0.2881	0.4626	0.125*	
C40	0.7207 (4)	−0.1233 (3)	0.5257 (3)	0.0870 (11)	
H40A	0.6651	−0.0449	0.5313	0.130*	
H40B	0.7689	−0.1558	0.5853	0.130*	
H40C	0.6552	−0.1644	0.5293	0.130*	

### Atomic displacement parameters (Å^2^)

**Table:**

	*U*^11^	*U*^22^	*U*^33^	*U*^12^	*U*^13^	*U*^23^
C1	0.059 (2)	0.082 (2)	0.072 (2)	−0.0160 (16)	−0.0187 (16)	−0.0192 (17)
C2	0.063 (2)	0.114 (3)	0.092 (3)	−0.034 (2)	−0.019 (2)	−0.021 (2)
C3	0.052 (2)	0.127 (3)	0.080 (2)	−0.026 (2)	−0.0044 (18)	−0.023 (2)
C4	0.0560 (19)	0.102 (3)	0.0614 (19)	−0.0108 (17)	−0.0111 (15)	−0.0175 (17)
C5	0.066 (2)	0.0655 (18)	0.0487 (16)	−0.0035 (15)	−0.0127 (14)	−0.0175 (14)
C6	0.091 (3)	0.069 (2)	0.0673 (19)	−0.0273 (18)	−0.0207 (19)	−0.0149 (16)
C7	0.098 (3)	0.081 (2)	0.082 (2)	−0.050 (2)	−0.015 (2)	−0.0135 (19)
C8	0.082 (2)	0.074 (2)	0.075 (2)	−0.0402 (18)	−0.0072 (18)	−0.0058 (18)
C9	0.070 (2)	0.0516 (17)	0.0559 (17)	−0.0165 (14)	−0.0066 (15)	−0.0040 (13)
C10	0.0499 (16)	0.0473 (14)	0.0472 (15)	−0.0010 (12)	−0.0148 (12)	−0.0046 (12)
C11	0.0484 (17)	0.0554 (16)	0.0547 (16)	−0.0031 (13)	−0.0208 (13)	−0.0065 (13)
C12	0.0517 (17)	0.0650 (17)	0.0479 (15)	−0.0064 (13)	−0.0155 (13)	−0.0103 (13)
C13	0.0552 (16)	0.0411 (13)	0.0448 (14)	−0.0081 (12)	−0.0141 (12)	0.0000 (12)
C14	0.0618 (18)	0.0518 (16)	0.0489 (16)	−0.0089 (14)	−0.0169 (14)	−0.0038 (13)
C15	0.0551 (16)	0.0451 (15)	0.0520 (16)	−0.0062 (12)	−0.0163 (13)	−0.0066 (14)
C16	0.0510 (16)	0.0478 (15)	0.0505 (15)	−0.0094 (12)	−0.0146 (13)	−0.0081 (14)
C17	0.0502 (15)	0.0398 (14)	0.0510 (15)	−0.0076 (11)	−0.0191 (12)	−0.0064 (12)
O18	0.0694 (13)	0.0396 (10)	0.0797 (13)	−0.0064 (8)	−0.0369 (10)	−0.0116 (9)
C19	0.078 (2)	0.079 (2)	0.0551 (17)	−0.0200 (17)	−0.0087 (16)	−0.0109 (16)
C20	0.071 (2)	0.0691 (19)	0.091 (2)	−0.0075 (15)	−0.0479 (18)	−0.0183 (17)
C21	0.065 (2)	0.085 (2)	0.077 (2)	−0.0254 (17)	−0.0344 (17)	0.0080 (18)
C22	0.071 (2)	0.111 (3)	0.101 (3)	−0.044 (2)	−0.033 (2)	0.015 (2)
C23	0.080 (2)	0.090 (2)	0.078 (2)	−0.038 (2)	−0.0195 (19)	0.0091 (19)
C24	0.076 (2)	0.0655 (19)	0.0596 (18)	−0.0212 (16)	−0.0258 (16)	0.0034 (15)
C25	0.0621 (19)	0.0519 (16)	0.0553 (16)	−0.0027 (14)	−0.0327 (14)	−0.0013 (13)
C26	0.0574 (19)	0.076 (2)	0.084 (2)	−0.0115 (16)	−0.0340 (17)	−0.0128 (17)
C27	0.0499 (19)	0.112 (3)	0.100 (3)	−0.0241 (19)	−0.0234 (18)	−0.015 (2)
C28	0.066 (2)	0.105 (3)	0.077 (2)	−0.041 (2)	−0.0083 (18)	−0.0102 (19)
C29	0.070 (2)	0.0683 (19)	0.0545 (17)	−0.0186 (15)	−0.0169 (15)	−0.0090 (14)
C30	0.0517 (16)	0.0496 (15)	0.0488 (15)	−0.0073 (12)	−0.0228 (12)	−0.0087 (12)
C31	0.0480 (16)	0.0565 (16)	0.0526 (15)	−0.0089 (13)	−0.0198 (13)	−0.0078 (13)
C32	0.0556 (17)	0.0497 (15)	0.0510 (15)	−0.0077 (13)	−0.0223 (13)	−0.0028 (13)
C33	0.0509 (16)	0.0525 (15)	0.0464 (15)	−0.0076 (12)	−0.0162 (13)	−0.0096 (12)
C34	0.0497 (16)	0.0564 (16)	0.0592 (16)	−0.0061 (13)	−0.0226 (13)	−0.0148 (14)
C35	0.0625 (18)	0.0565 (16)	0.0545 (16)	−0.0120 (13)	−0.0278 (14)	−0.0033 (13)
C36	0.0665 (18)	0.0537 (16)	0.0572 (16)	−0.0168 (13)	−0.0281 (14)	−0.0043 (13)
C37	0.0611 (18)	0.0561 (17)	0.0563 (16)	−0.0114 (13)	−0.0347 (14)	−0.0032 (13)
O38	0.0616 (12)	0.0555 (11)	0.0874 (14)	−0.0091 (9)	−0.0470 (11)	−0.0072 (10)
C39	0.121 (3)	0.0475 (17)	0.088 (2)	−0.0088 (17)	−0.059 (2)	−0.0052 (16)
C40	0.089 (2)	0.103 (3)	0.061 (2)	−0.026 (2)	−0.0264 (18)	0.0053 (18)

### Geometric parameters (Å, °)

**Table:**

C1—C2	1.348 (4)	C21—C22	1.341 (4)
C1—C11	1.407 (4)	C21—C31	1.397 (4)
C1—H1	0.9300	C21—H21	0.9300
C2—C3	1.397 (5)	C22—C23	1.388 (4)
C2—H2	0.9300	C22—H22	0.9300
C3—C4	1.343 (5)	C23—C24	1.325 (4)
C3—H3	0.9300	C23—H23	0.9300
C4—C12	1.409 (4)	C24—C32	1.404 (4)
C4—H4	0.9300	C24—H24	0.9300
C5—C12	1.373 (4)	C25—C32	1.375 (4)
C5—C14	1.378 (4)	C25—C34	1.375 (4)
C5—H5	0.9300	C25—H25	0.9300
C6—C7	1.334 (5)	C26—C27	1.336 (5)
C6—C14	1.417 (4)	C26—C34	1.401 (4)
C6—H6	0.9300	C26—H26	0.9300
C7—C8	1.391 (5)	C27—C28	1.399 (5)
C7—H7	0.9300	C27—H27	0.9300
C8—C9	1.346 (4)	C28—C29	1.333 (4)
C8—H8	0.9300	C28—H28	0.9300
C9—C13	1.400 (4)	C29—C33	1.419 (4)
C9—H9	0.9300	C29—H29	0.9300
C10—C11	1.398 (4)	C30—C33	1.390 (4)
C10—C13	1.407 (4)	C30—C31	1.397 (4)
C10—C15	1.426 (4)	C30—C35	1.427 (3)
C11—C12	1.416 (4)	C31—C32	1.421 (3)
C13—C14	1.411 (4)	C33—C34	1.420 (3)
C15—C16	1.185 (4)	C35—C36	1.180 (3)
C16—C17	1.453 (4)	C36—C37	1.466 (3)
C17—O18	1.425 (3)	C37—O38	1.426 (3)
C17—C20	1.498 (4)	C37—C40	1.479 (4)
C17—C19	1.500 (4)	C37—C39	1.500 (4)
O18—H18	0.8200	O38—H38	0.8200
C19—H19A	0.9600	C39—H39A	0.9600
C19—H19B	0.9600	C39—H39B	0.9600
C19—H19C	0.9600	C39—H39C	0.9600
C20—H20A	0.9600	C40—H40A	0.9600
C20—H20B	0.9600	C40—H40B	0.9600
C20—H20C	0.9600	C40—H40C	0.9600
			
C2—C1—C11	121.2 (3)	C22—C21—C31	121.3 (3)
C2—C1—H1	119.4	C22—C21—H21	119.4
C11—C1—H1	119.4	C31—C21—H21	119.4
C1—C2—C3	120.7 (3)	C21—C22—C23	121.0 (3)
C1—C2—H2	119.7	C21—C22—H22	119.5
C3—C2—H2	119.7	C23—C22—H22	119.5
C4—C3—C2	119.9 (3)	C24—C23—C22	119.7 (3)
C4—C3—H3	120.0	C24—C23—H23	120.1
C2—C3—H3	120.0	C22—C23—H23	120.1
C3—C4—C12	121.4 (3)	C23—C24—C32	122.0 (3)
C3—C4—H4	119.3	C23—C24—H24	119.0
C12—C4—H4	119.3	C32—C24—H24	119.0
C12—C5—C14	121.9 (3)	C32—C25—C34	122.5 (2)
C12—C5—H5	119.1	C32—C25—H25	118.8
C14—C5—H5	119.1	C34—C25—H25	118.8
C7—C6—C14	121.9 (3)	C27—C26—C34	121.5 (3)
C7—C6—H6	119.1	C27—C26—H26	119.2
C14—C6—H6	119.1	C34—C26—H26	119.2
C6—C7—C8	119.9 (3)	C26—C27—C28	120.6 (3)
C6—C7—H7	120.0	C26—C27—H27	119.7
C8—C7—H7	120.0	C28—C27—H27	119.7
C9—C8—C7	120.5 (3)	C29—C28—C27	119.9 (3)
C9—C8—H8	119.7	C29—C28—H28	120.0
C7—C8—H8	119.7	C27—C28—H28	120.0
C8—C9—C13	121.4 (3)	C28—C29—C33	121.9 (3)
C8—C9—H9	119.3	C28—C29—H29	119.1
C13—C9—H9	119.3	C33—C29—H29	119.1
C11—C10—C13	120.4 (2)	C33—C30—C31	121.1 (2)
C11—C10—C15	119.5 (3)	C33—C30—C35	119.7 (3)
C13—C10—C15	120.0 (2)	C31—C30—C35	119.2 (2)
C10—C11—C1	122.4 (3)	C30—C31—C21	123.2 (2)
C10—C11—C12	119.4 (3)	C30—C31—C32	119.1 (2)
C1—C11—C12	118.2 (3)	C21—C31—C32	117.7 (3)
C5—C12—C4	121.9 (3)	C25—C32—C24	122.7 (2)
C5—C12—C11	119.5 (3)	C25—C32—C31	119.0 (3)
C4—C12—C11	118.7 (3)	C24—C32—C31	118.3 (3)
C9—C13—C10	122.5 (3)	C30—C33—C29	123.4 (2)
C9—C13—C14	118.4 (3)	C30—C33—C34	119.1 (2)
C10—C13—C14	119.0 (3)	C29—C33—C34	117.5 (3)
C5—C14—C13	119.8 (3)	C25—C34—C26	122.3 (3)
C5—C14—C6	122.4 (3)	C25—C34—C33	119.2 (2)
C13—C14—C6	117.8 (3)	C26—C34—C33	118.6 (3)
C16—C15—C10	176.9 (3)	C36—C35—C30	176.2 (3)
C15—C16—C17	177.3 (3)	C35—C36—C37	178.5 (3)
O18—C17—C16	108.37 (19)	O38—C37—C36	107.7 (2)
O18—C17—C20	106.5 (2)	O38—C37—C40	110.3 (2)
C16—C17—C20	109.6 (2)	C36—C37—C40	110.7 (2)
O18—C17—C19	109.2 (2)	O38—C37—C39	105.0 (2)
C16—C17—C19	110.9 (2)	C36—C37—C39	110.5 (2)
C20—C17—C19	112.1 (2)	C40—C37—C39	112.4 (3)
C17—O18—H18	109.5	C37—O38—H38	109.5
C17—C19—H19A	109.5	C37—C39—H39A	109.5
C17—C19—H19B	109.5	C37—C39—H39B	109.5
H19A—C19—H19B	109.5	H39A—C39—H39B	109.5
C17—C19—H19C	109.5	C37—C39—H39C	109.5
H19A—C19—H19C	109.5	H39A—C39—H39C	109.5
H19B—C19—H19C	109.5	H39B—C39—H39C	109.5
C17—C20—H20A	109.5	C37—C40—H40A	109.5
C17—C20—H20B	109.5	C37—C40—H40B	109.5
H20A—C20—H20B	109.5	H40A—C40—H40B	109.5
C17—C20—H20C	109.5	C37—C40—H40C	109.5
H20A—C20—H20C	109.5	H40A—C40—H40C	109.5
H20B—C20—H20C	109.5	H40B—C40—H40C	109.5
			
C11—C1—C2—C3	−0.5 (5)	C31—C21—C22—C23	−1.6 (6)
C1—C2—C3—C4	0.0 (6)	C21—C22—C23—C24	1.1 (6)
C2—C3—C4—C12	0.8 (5)	C22—C23—C24—C32	−0.2 (5)
C14—C6—C7—C8	−1.0 (5)	C34—C26—C27—C28	−0.1 (5)
C6—C7—C8—C9	−1.2 (5)	C26—C27—C28—C29	−0.2 (6)
C7—C8—C9—C13	2.5 (5)	C27—C28—C29—C33	1.2 (5)
C13—C10—C11—C1	177.9 (2)	C33—C30—C31—C21	−179.8 (3)
C15—C10—C11—C1	1.6 (4)	C35—C30—C31—C21	1.7 (4)
C13—C10—C11—C12	−0.6 (4)	C33—C30—C31—C32	−1.5 (4)
C15—C10—C11—C12	−176.9 (2)	C35—C30—C31—C32	−179.9 (2)
C2—C1—C11—C10	−178.2 (3)	C22—C21—C31—C30	179.6 (3)
C2—C1—C11—C12	0.3 (4)	C22—C21—C31—C32	1.3 (5)
C14—C5—C12—C4	−178.1 (3)	C34—C25—C32—C24	−179.9 (3)
C14—C5—C12—C11	0.6 (4)	C34—C25—C32—C31	−0.6 (4)
C3—C4—C12—C5	177.6 (3)	C23—C24—C32—C25	179.3 (3)
C3—C4—C12—C11	−1.0 (4)	C23—C24—C32—C31	−0.1 (5)
C10—C11—C12—C5	0.3 (4)	C30—C31—C32—C25	1.8 (4)
C1—C11—C12—C5	−178.2 (3)	C21—C31—C32—C25	−179.8 (3)
C10—C11—C12—C4	179.0 (2)	C30—C31—C32—C24	−178.9 (2)
C1—C11—C12—C4	0.5 (4)	C21—C31—C32—C24	−0.4 (4)
C8—C9—C13—C10	175.6 (3)	C31—C30—C33—C29	179.2 (3)
C8—C9—C13—C14	−1.7 (4)	C35—C30—C33—C29	−2.4 (4)
C11—C10—C13—C9	−177.3 (2)	C31—C30—C33—C34	0.0 (4)
C15—C10—C13—C9	−1.0 (4)	C35—C30—C33—C34	178.4 (2)
C11—C10—C13—C14	0.0 (3)	C28—C29—C33—C30	178.8 (3)
C15—C10—C13—C14	176.3 (2)	C28—C29—C33—C34	−2.0 (4)
C12—C5—C14—C13	−1.2 (4)	C32—C25—C34—C26	179.4 (3)
C12—C5—C14—C6	177.5 (3)	C32—C25—C34—C33	−0.9 (4)
C9—C13—C14—C5	178.3 (2)	C27—C26—C34—C25	179.0 (3)
C10—C13—C14—C5	0.9 (3)	C27—C26—C34—C33	−0.7 (4)
C9—C13—C14—C6	−0.4 (3)	C30—C33—C34—C25	1.2 (4)
C10—C13—C14—C6	−177.8 (2)	C29—C33—C34—C25	−178.0 (3)
C7—C6—C14—C5	−177.0 (3)	C30—C33—C34—C26	−179.1 (3)
C7—C6—C14—C13	1.7 (4)	C29—C33—C34—C26	1.7 (4)

### Hydrogen-bond geometry (Å, °)

**Table:**

*D*—H···*A*	*D*—H	H···*A*	*D*···*A*	*D*—H···*A*
O18—H18···O38^i^	0.82	2.01	2.726 (3)	145
O38—H38···O18^ii^	0.82	2.06	2.766 (3)	145

Symmetry codes:  (i) −*x*+1, −*y*+1, −*z*+1; (ii) *x*+1, *y*−1, *z*.

### Table 2 C—H···π interactions (Å,°).

**Table:**

X	H	J	H···J	X···J	X-I···J
C19	H19C	Cg1^iii^	2.87	3.810 (4)	167
C20	H20B	Cg2^iii^	2.76	3.703 (4)	168
C24	H24	Cg2^iv^	2.64	3.472 (3)	149
C25	H25	Cg1^iv^	2.92	3.794 (3)	157

Symmetry codes: (iii) 1-y, -y, 1-z; (iv) 1-x, 1-y, -z. Cg1 is the centroid of the C5/C10–C14 ring and Cg2 is the centroid of the C1–C4/C12/C11 ring.

### Table 3 π-π interactions (Å,°).

**Table:**

CgI	CgJ	Cg···Cg	Dihedral angle	Interplanar distance	Offset
3	4^v^	3.794 (2)	1.2	3.370 (2)	1.336 (2)
4	3^v^	3.794 (2)	1.2	3.404 (2)	1.464 (2)

Symmetry code: (v) 1-x, -y, -z. Cg3 is the centroid of the C25/30–C34 ring and Cg4 is the centroid of the C26-C29/C33/C34 ring. The dihedral angle is that between the planes of the rings CgI and CgJ. The interplanar distance is the perpendicular distance of CgI from ring J. The offset is the perpendicular distance of ring I from ring J.
